# A case of late lymph node metastasis after three endoscopic mucosal resections of intramucosal gastric cancers

**DOI:** 10.1186/1477-7819-12-339

**Published:** 2014-11-11

**Authors:** Eisuke Booka, Tsunehiro Takahashi, Kazunori Tokizawa, Yusuke Uchi, Akihiko Okamura, Kazumasa Fukuda, Rieko Nakamura, Norihito Wada, Hirofumi Kawakubo, Yoshiro Saikawa, Tai Omori, Hiroya Takeuchi, Aya Sasaki, Shuji Mikami, Koichiro Kumai, Kaori Kameyama, Yuko Kitagawa

**Affiliations:** Department of Surgery, Keio University School of Medicine, 35 Shinanomachi, Shinjuku-ku, Tokyo, 160-8582 Japan; Division of Diagnostic Pathology, Keio University Hospital, 35 Shinanomachi, Shinjuku-ku, Tokyo, 160-8582 Japan; Department of Surgery, Hino Municipal Hospital, 4-3-1 Tamadaira, Hino, Tokyo, 191-0062 Japan

**Keywords:** Endoscopic mucosal resection (EMR), Intramucosal gastric cancer, Lymph node metastasis, Piecemeal resection

## Abstract

We describe a patient with solitary lymph node (LN) metastasis after three endoscopic mucosal resections (EMRs) in which a gastrointestinal stromal tumor was difficult to differentiate from the carcinoid and lymphoma tumors. A 77-year-old man underwent three EMRs at 62, 72, and 75 years of age, and all resections were determined to be curative. However, 2 years after the last EMR, screening abdominal ultrasonography detected a 20-mm solitary tumor at the lesser curvature of the upper stomach. Laparoscopic tumor resection confirmed the pathological diagnosis. Intraoperative pathological diagnosis showed that the adenocarcinoma was compatible with recurrence of gastric cancer; thus, total gastrectomy with D1 lymphadenectomy was performed. Metastasis was not recognized by pathological examination but was detected by preoperative radiological examinations of the LN. We report a rare recurrence case after several EMRs of intramucosal gastric cancers.

## Background

In Japan, the Gastric Cancer Treatment Guidelines (GCTGs) (ver. 3) define absolute indications for endoscopic resection (ER), which include ≤20-mm intramucosal differentiated cancers without an ulcer [1]. Curative resection is restricted to en bloc resection in the latest guideline [1]. However, before the GCTGs were established, piecemeal resection was considered to be curative when the specimen was completely reconstructed and showed negative lymphovascular invasions and horizontal/vertical margins [2]. We experienced a rare late recurrence case of lymph node (LN) metastasis after endoscopic mucosal resections (EMRs) including a piecemeal resection that were curative on the basis of the GCTGs criteria at that time [1–3].

## Case presentation

**Figure 1:**
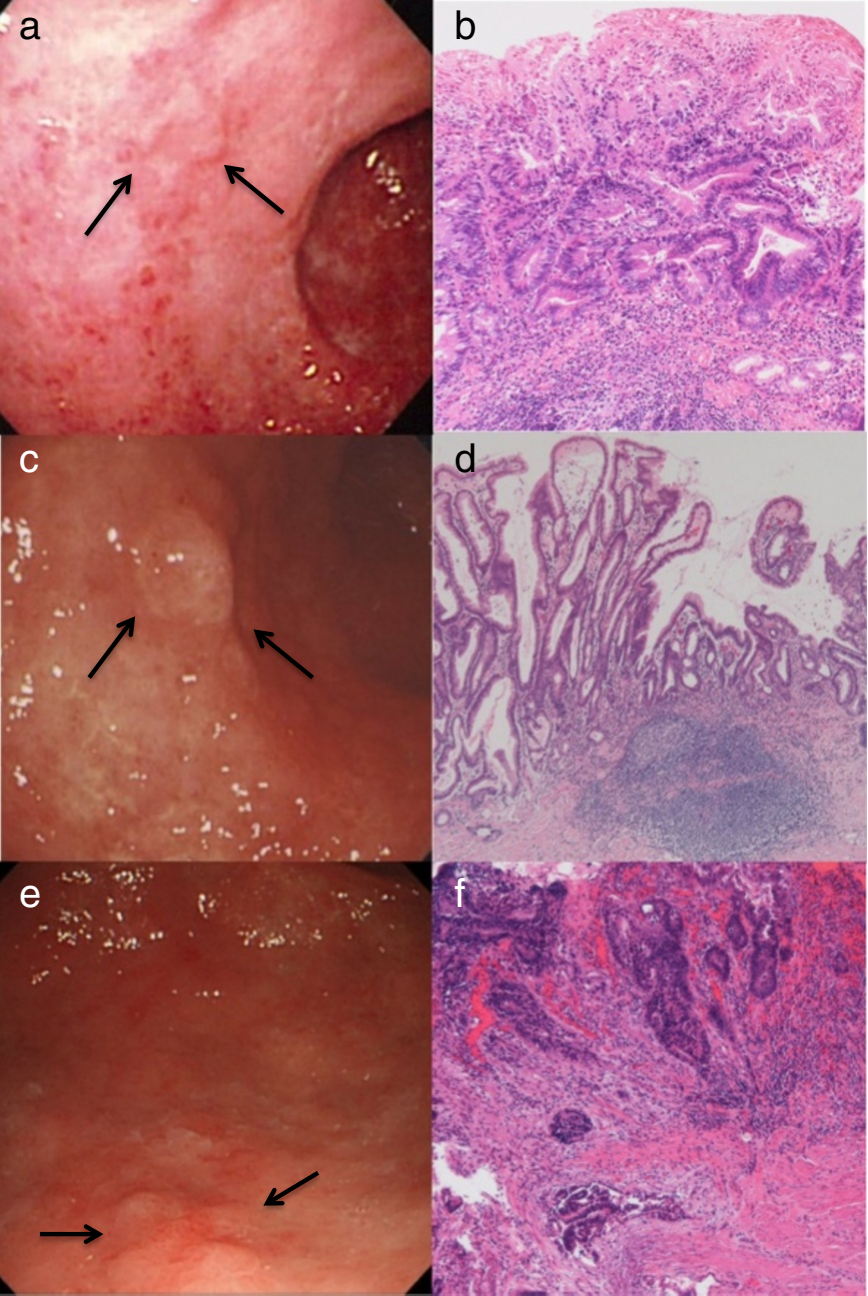
**Endoscopic and pathological findings of three endoscopic mucosal resections (EMRs). (a, b)** Endoscopy showed a type 0-IIc lesion 10 mm in size without an ulcer on the lesser curvature of the antrum in 1998 **(a)**. Pathological examination revealed moderately differentiated adenocarcinoma in the intramucosal proximal portion of the lesion **(b)**. **(c, d)** Endoscopy showed a type 0-IIa lesion 12 mm in size without an ulcer on the anterior wall near the pylorus in 2008 **(c)**. Pathological examination revealed predominantly moderately differentiated adenocarcinoma with papillary adenocarcinoma component in the intramucosal proximal portion of the lesion **(d)**. **(e, f)** Endoscopy showed a type 0-IIa + IIc lesion 7 mm in size without an ulcer on the greater curvature of the antrum in 2011 **(e)**. Pathological examination revealed moderately differentiated adenocarcinoma remaining in the muscularis mucosa **(f)**.

**Figure 2:**
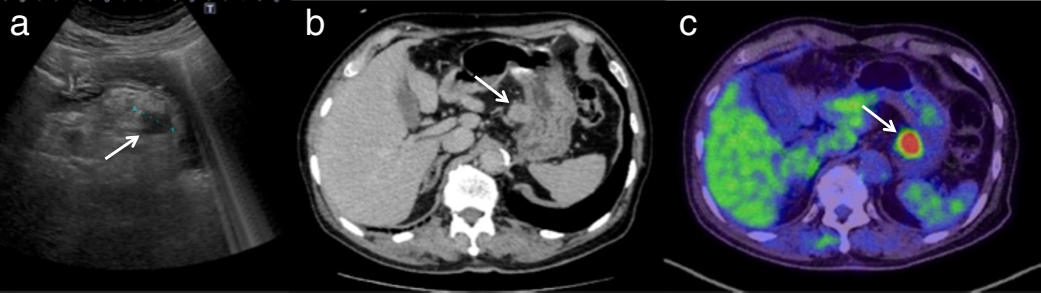
**Abdominal ultrasonography (AUS), computed tomography (CT) and positron emission tomography-computed tomography (PET-CT) findings of the tumor. (a)** Abdominal ultrasonography showed the tumor 20 mm in size along the lesser curvature of the stomach. **(b)** CT scan showed the tumor 20 mm in size at the same lesion. **(c)** PET-CT showed an FDG hot uptake at the same lesion.

**Figure 3:**
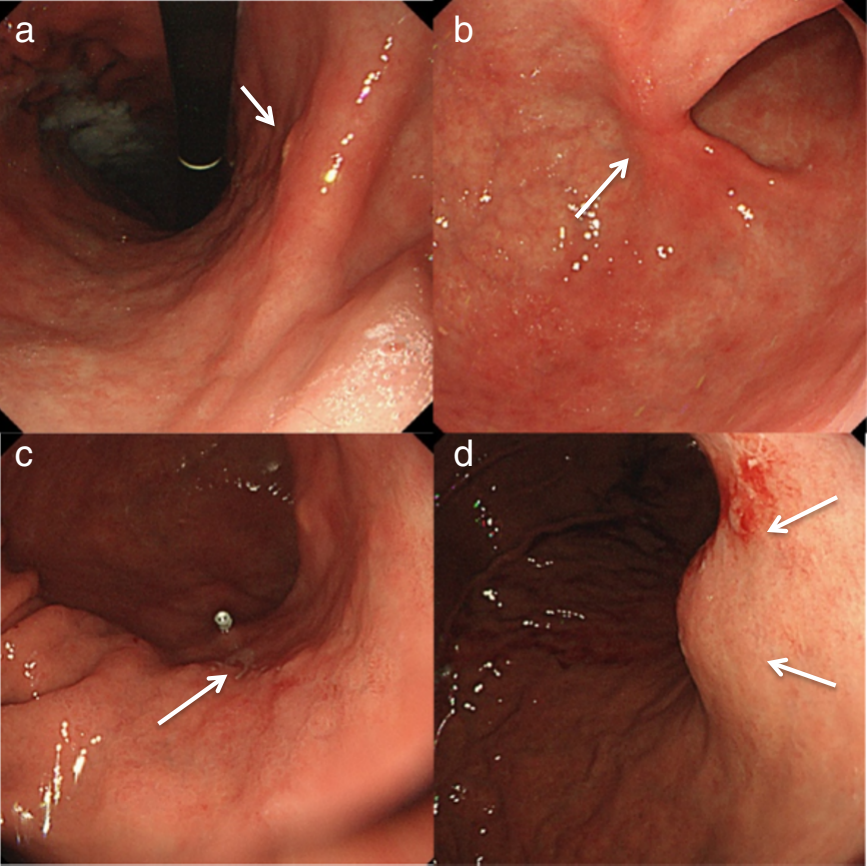
**Endoscopic findings of three endoscopic mucosal resection (EMR) scars and an elevated lesion.** Endoscopy showed the first EMR scar on the lesser curvature of the antrum **(a)**, the second EMR scar on the anterior wall near the pylorus **(b)**, last EMR scar on the greater curvature of the antrum **(c)**, and an elevated lesion displaced from the outside at the lesser curvature of the upper stomach **(d)**.

**Figure 4:**
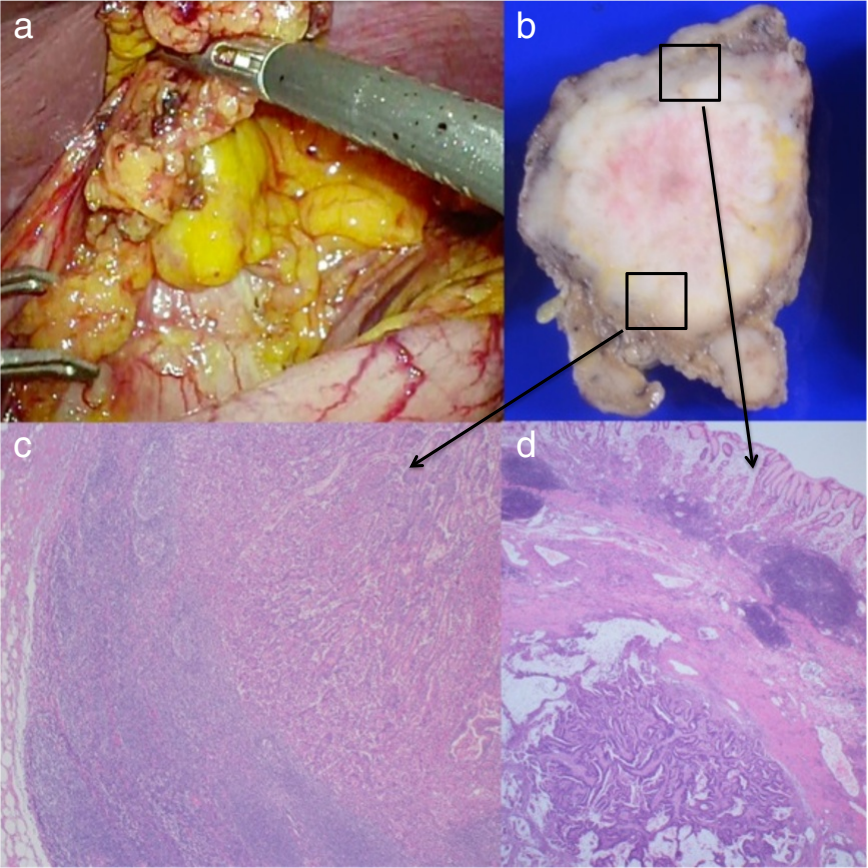
**Intraoperative laparoscopic and postoperative pathological findings. (a)** Laparoscopic findings of an enlarged lymph node (LN) located along the lesser curvature of the upper stomach. **(b)** Resected tumor with LN metastasis invading the stomach wall. **(c, d)** Postoperative pathological examination revealed that the resected LN was compatible with metastasis of moderately differentiated adenocarcinoma **(c)** and had invaded the stomach wall **(d)**.

A 62-year-old man was admitted in 1998 because early gastric cancer (EGC) was detected by annual screening endoscopy. There was no specific finding in the physical examination or laboratory data. He had no medical history of malignant tumors. The lesion was a 10-mm type 0-IIc moderately differentiated adenocarcinoma without an ulcer located at the lesser curvature of the antrum (Figure 1a). We diagnosed that this lesion had a negligible risk of LN metastasis, and ER was indicated [4]. We explained to the patient that EMR was an investigational treatment at that time, and he chose to receive EMR instead of surgery. EMR using piecemeal resection was performed, which was curative macroscopically. The specimens were completely reconstructed, and pathological examination confirmed a 10-mm type 0-IIc moderately differentiated adenocarcinoma, without an ulcer (Figure 1b). The tumor was confined to the mucosa with negative lymphovascular invasions and horizontal/vertical margins, which indicated that the resection was curative according to the Japanese 13th edition of the Classification of Gastric Carcinoma [2]. We performed endoscopy at 1, 3, and 6 months after EMR to check for local recurrence, and every biopsy of the EMR scars revealed no malignancy. Thereafter, we performed follow-up endoscopy and abdominal ultrasonography (AUS) to check locoregional or distant metastasis every year, and no recurrence was detected. After 10 years, another lesion was detected by endoscopy in 2008 when the patient was 72 years old. The lesion was a 12-mm type 0-IIa moderately differentiated adenocarcinoma without an ulcer located at the anterior wall near the pylorus (Figure 1c). En bloc EMR was performed; pathological examination revealed a 12-mm type 0-IIa lesion without an ulcer that was predominantly a moderately differentiated adenocarcinoma with papillary adenocarcinoma components (Figure 1d). The tumor was confined to the mucosa with negative lymphovascular invasions and horizontal/vertical margins, which indicated that the resection was curative according to the GCTG (ver. 2) [3]. Thereafter, we performed follow-up endoscopy and AUS every year; recurrence was not observed, but a nonrecurrent lesion was detected in 2011 when the patient was 75 years old. The lesion was a 7-mm type 0-IIa + IIc moderately differentiated adenocarcinoma without an ulcer, located at the greater curvature of the antrum (Figure 1e). En bloc EMR was performed; pathological examination revealed a 1-mm moderately differentiated adenocarcinoma without an ulcer (Figure 1f). The tumor was confined to the mucosa with negative lymphovascular invasions and horizontal/vertical margins. However, the tumor seemed to invade into the muscularis mucosa, which indicated that the resection was curative (GCTGs, ver. 3) [1]. Although subsequent endoscopy did not detect recurrence, a 20-mm tumor was detected along the lesser curvature of the stomach by screening AUS in 2013 when the patient was 77 years old (Figure 2a). We performed computed tomography (CT) and positron emission tomography (PET)-CT. CT showed a 20-mm tumor along the lesser curvature of the stomach and no other metastatic lesion (Figure 2b). PET-CT showed 18 F-fluorodeoxyglucose hot uptake in the same lesion (Figure 2c). We performed endoscopy to check for new lesions or local recurrence. The endoscopy showed three lesions on EMR scars and an elevated lesion displaced from the outside at the lesser curvature of the upper stomach that had not been detected at the last endoscopy (Figure 3a-d). We performed biopsies of each EMR scar and the elevated lesion, but no malignancy was identified. Gastrointestinal stromal tumor (GIST), carcinoid tumor, and lymphoma were considered in the differential diagnosis along with LN metastasis. Although endoscopic ultrasound (EUS)-guided fine-needle aspiration (FNA) was considered to determine a pathological diagnosis, EUS-FNA was not performed because of the risk of dissemination. We performed a laparoscopic total excisional biopsy to resect the tumor, and the intraoperative frozen section indicated LN metastasis of the adenocarcinoma (Figure 4a). Because this LN was recognized as #3a LN with stomach invasion, the operation was converted to an open standard total gastrectomy with D1 LN dissection. Postoperative pathological examination indicated that the resected LN was a metastasis of the moderately differentiated adenocarcinoma with stomach wall invasion (Figure 4b-d). Additionally, no remnant or recurrent malignancy was detected at any stomach EMR site, and no other metastasis was found among the 52 other LNs retrieved.

### Discussion

Currently, indications for endoscopic treatment of gastric cancer are based on retrospective analysis of the incidence of lymph node metastasis [5]. The GCTGs (ver. 3) define absolute indications for ER, which include ≤20-mm intramucosal differentiated cancers without an ulcer [1]. When the first EMR was performed, there were no guidelines for gastric cancer treatment, and definitive indications for endoscopic treatment had not been established. However, in our case, a retrospective analysis showed that there was a negligible risk of LN metastasis, and the lesion was considered to be suitable for endoscopic treatment [4]. The first EMR involving piecemeal resection initially considered to be curative was later considered to have been an incomplete resection [1, 2]. However, incomplete resection only caused by piecemeal resection need not always require additional surgery [1]. Horiki *et al*. reported that piecemeal EMR elevated the risk of local recurrence but not of LN metastasis [6]. Because evaluation of horizontal margins is extremely important in EMR, en bloc resection is necessary, and endoscopic submucosal dissection (ESD) would be superior for the evaluation of horizontal margins. Although ESD has been widely performed, only a few cases of metastasis have been reported after endoscopic curative resection of EGCs that met the indication criteria [7–9]. Because cases of LN metastasis after curative ERs and solitary LN metastasis are rare, we considered GIST, carcinoid tumor, and lymphoma in the differential diagnosis along with LN metastasis. Although it might be possible to confirm the pathological diagnosis by EUS-FNA, its sensitivity and specificity in patients with lymphadenopathy suspected of recurrent malignancy is low and has a risk of dissemination in the case of extrinsic tumors. Hence, we performed laparoscopic surgery to resect the tumor as a total excisional biopsy [10]. In the present case, we performed three EMRs, and every pathological examination revealed moderately differentiated adenocarcinoma, which indicates that it is difficult to pathologically predict the primary tumor of metastatic LN. It was possible that LN metastasis rose from the last EMR lesion because the tumor seemed to invade into the muscularis mucosa. However, in gastric cancers, the risk of LN metastasis from the muscularis mucosa is lower than that of esophageal cancers [1, 11, 12]. Given that the first EMR was a piecemeal resection, this lesion was thought to be the original one. However, if this lesion caused the LN metastasis, this case is especially unique because the metastasis was detected 15 years after the EMR. Although the piecemeal resection was considered to have elevated the risk of local recurrence but not of LN metastasis, it was considered to have contributed to the very slow LN metastasis [6]. Our case might represent a limitation of ER in evaluating LN status. This viewpoint is consistent with a recent guideline in which piecemeal resection is considered to be incomplete [1].

## Conclusions

This case presents a rare case of late LN metastasis after EMR based on the indications for endoscopic treatment of gastric cancer. This case might represent a limitation of ER in evaluating LN status. Because piecemeal resection has a risk of not only local recurrence but also of LN metastasis, en block resection is required, and careful follow-up is mandatory.

## Consent

Written informed consent for publication of this case report and any accompanying images was obtained from the patient. A copy of the written consent is available for review by the Editor-in-Chief of this journal.

## Abbreviations

AUS: abdominal ultrasonography
CT: computed tomography
EGC: early gastric cancer
EMR: endoscopic mucosal resection
ER: endoscopic resection
ESD: endoscopic submucosal dissection
EUS: endoscopic ultrasound
FNA: fine-needle aspiration
GCTGs: Gastric Cancer Treatment Guidelines
GIST: gastrointestinal stromal tumor
LN: lymph node
PET: positron emission tomography.
